# Natural Regenerative Hydrogels for Wound Healing

**DOI:** 10.3390/gels10090547

**Published:** 2024-08-23

**Authors:** Mariana Chelu, Jose M. Calderon Moreno, Adina Magdalena Musuc, Monica Popa

**Affiliations:** “Ilie Murgulescu” Institute of Physical Chemistry, 202 Spl. Independentei, 060021 Bucharest, Romania; mchelu@icf.ro (M.C.); amusuc@icf.ro (A.M.M.)

**Keywords:** regenerative hydrogels, tissue engineering, wound healing, extracellular matrix, antibacterial activity, biocompatibility, biodegradability, glycosaminoglycans, polysaccharide gums, cross-linking mechanisms

## Abstract

Regenerative hydrogels from natural polymers have come forth as auspicious materials for use in regenerative medicine, with interest attributed to their intrinsic biodegradability, biocompatibility, and ability to reassemble the extracellular matrix. This review covers the latest advances in regenerative hydrogels used for wound healing, focusing on their chemical composition, cross-linking mechanisms, and functional properties. Key carbohydrate polymers, including alginate, chitosan, hyaluronic acid, and polysaccharide gums, including agarose, carrageenan, and xanthan gum, are discussed in terms of their sources, chemical structures and specific properties suitable for regenerative applications. The review further explores the categorization of hydrogels based on ionic charge, response to physiological stimuli (i.e., pH, temperature) and particularized roles in wound tissue self-healing. Various methods of cross-linking used to enhance the mechanical and biological performance of these hydrogels are also examined. By highlighting recent innovations and ongoing challenges, this article intends to give a detailed understanding of natural hydrogels and their potential to revolutionize regenerative medicine and improve patient healing outcomes.

## 1. Introduction

Regenerative hydrogels are complex biomaterials designed to aid in successful wound regeneration and healing of multiple tissue types. These hydrogels are often based on natural carbohydrate polymers, including polysaccharides like cellulose, chitosan, alginate, and hyaluronic acid, due to biodegradability, biocompatibility and functional versatility [1,2,3].

**Figure 1:**
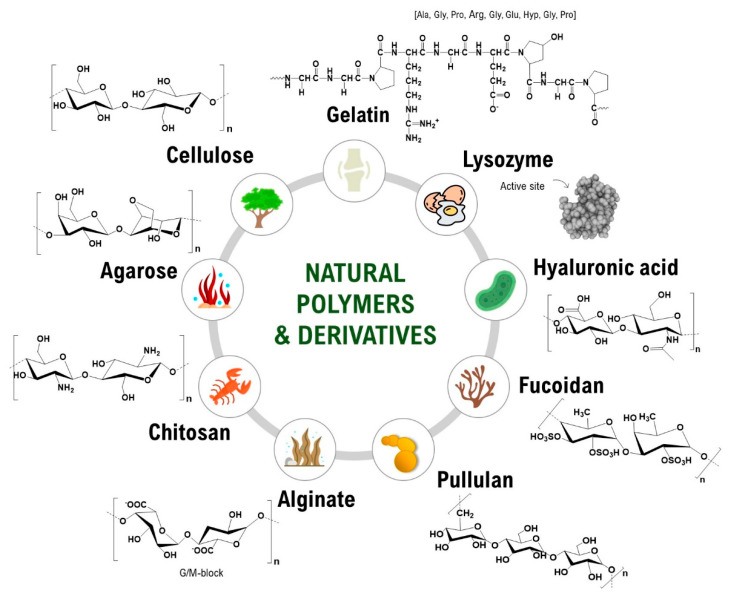
Natural carbohydrate polymers used for natural hydrogels [4].

Natural carbohydrate polymers (NCPs) are long-chain polymers of sugar monomers connected by glycosidic bonds. Abundant in nature and renewable, they are non-toxic, generally non-immunogenic and possess advantageous intrinsic properties as hydrogel foundational material favorable for biomedical applications; they have a high affinity for water hydrophilicity, biocompatibility and biodegradability. Moreover, their chemical structure allows for various modifications to tailor their properties to the desired use (Figure 1) [4,5,6,7]. NCPs used in regenerative hydrogels (i.e., in wound dressings) include those from plants (cellulose), seaweed (alginate), or animal tissues (chitosan, obtained from chitin, found in the exoskeleton of crustaceans; hyaluronic acid, obtained from rooster combs and bovine synovial fluids) [8]. Applications in regenerative medicine include wound healing, as hydrogels provide a moist environment conducive to healing and can be loaded with antimicrobial agents or growth factors to enhance tissue repair; tissue engineering as scaffolds that mimic the natural extracellular matrix and support cell adhesion, proliferation, and differentiation; drug delivery because hydrogels can encapsulate drugs and provide controlled release, useful for localized treatment and reducing systemic side effects; and cartilage and bone regeneration, as hydrogels can be used to deliver cells and growth factors to repair cartilage and bone defects (hyaluronic acid-based hydrogels are considered particularly effective in cartilage regeneration) [9,10,11,12,13]. This review covers the latest advances in regenerative hydrogels used for wound healing and intends to highlight the potential of natural hydrogels to revolutionize regenerative medicine.

## 2. Natural Regenerative Hydrogels

**Figure 2:**
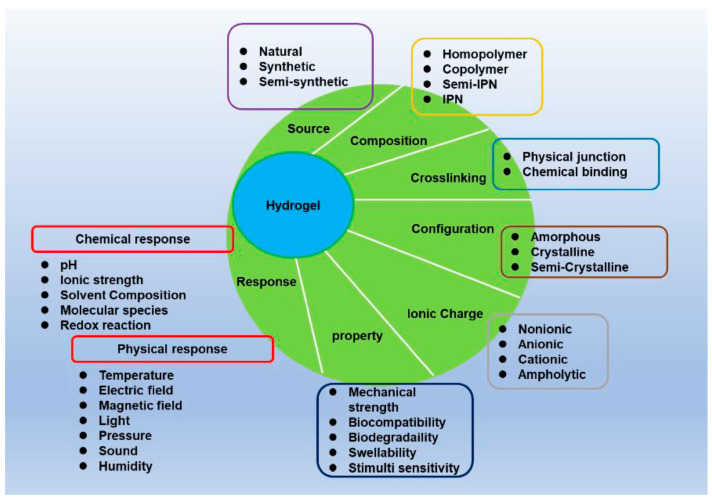
The main characteristics of hydrogels [14].

Natural hydrogels offer a diverse range of properties based on the ionic charge that makes them suitable for biomedical applications in tissue engineering, drug delivery, and wound healing (Figure 2) [14].

*Anionic* natural hydrogels carry negatively charged groups (alginate or hyaluronic acid contain carboxylate groups; pectin contains carboxyl groups), which allow them to interact with positively charged molecules and ions, serving to create a moist environment that promotes cell migration or drug delivery for positively charged drugs [15].

*Cationic* natural hydrogels, such as chitosan, carry positively charged groups, enabling interactions with negatively charged molecules and ions, and are generally suitable for antimicrobial applications [16,17].

*Ampholytic* or *zwitterionic* natural hydrogels contain both positively and negatively charged groups, allowing them to exhibit unique properties such as reduced protein adsorption and high biocompatibility [18].

Finally, non-ionic natural hydrogels (agarose, gelatin) do not carry any net charge, provide general biocompatibility and are mostly used in tissue engineering, while anionic and cationic hydrogels allow for specific interactions with charged molecules and cells [19].

Ampholytic hydrogels offer unique properties for specialized uses, and composite hydrogels enable the combination of different functionalities to meet complex biomedical needs [20].

Regenerative hydrogels can be assembled through various *cross-linking methods*: *physical* (ionic interactions, hydrogen bonding, and hydrophobic interactions) and *chemical* (covalent bonding through reactions like esterification, amidation, or click chemistry). Each method has its advantages and specific applications in forming regenerative hydrogels (Table 1) [21].

Physical cross-linking involves non-covalent interactions to form hydrogels: hydrogen bonding, ionic and hydrophobic interactions, and crystallization [22].

Ionic cross-linking involves the interactions between oppositely charged ions. This method is common for polymers like alginate, which gellifies in the presence of divalent cations (e.g., Ca^2+^) [23].

Hydrogels can also be formed through extensive hydrogen bonding between polymer chains, often seen in natural polymers such as agarose and gelatin, by cooling a hot aqueous solution, where hydrogen bonds stabilize the gel network [24].

Hydrophobic segments of amphiphilic polymers can aggregate in aqueous media, leading to the formation of hydrogels through hydrophobic interactions, often temperature-sensitive [25].

Crystallizable segments within polymers can form physical cross-links through crystallization. This method is temperature-dependent, and it is reversible. Physical methods are generally reversible and also do not require chemical additives, which makes them desirable for applications where biocompatibility is critical.

Chemical cross-linking involves the formation of covalent chemical bonds between polymer chains. As a result, a stable and often irreversible hydrogel network is formed [26]. This method is adequate for precise control over the hydrogel’s mechanical properties and degradation rates. Free radical polymerization involves the formation of radicals that initiate polymerization of monomers and polymers cross-linking [27].

Click chemistry refers to a group of highly efficient, specific reactions that are biorthogonal (i.e., they do not interfere with biological processes). The most common click reaction used in hydrogel formation is the azide-alkyne cycloaddition, used, for example, in azide-functionalized hyaluronic acid hydrogels [28].

Schiff base formation involves the reaction between an aldehyde or ketone group and an amine, giving, as a result, an imine covalent bond. This reaction can be reversible under physiological conditions, making it suitable for dynamic hydrogels, for example, in chitosan hydrogels cross-linked with oxidized dextran [29].

Enzymatic cross-linking makes use of enzymes in order to catalyze the formation of covalent chemical bonds between chains of polymers. This method is often highly specific and conducted under mild conditions. One example is gelatin hydrogels cross-linked using transglutaminase [30].

Also, mall bifunctional molecules can act as cross-linkers, reacting with functional groups on polymer chains to form covalent bonds, for example, glutaraldehyde cross-linking of gelatin or collagen [31].

Chemical cross-linking offers stability and control, ideal for applications demanding precise mechanical properties and durability. The choice of cross-linking method depends on the particular needs of the intended biomedical application [32].

Natural regenerative hydrogels can be categorized *based on the composition of their polymer chains*: *homopolymeric*, *copolymeric*, and *hybrid* (multipolymeric). This classification helps in understanding their structural diversity, functional properties, and suitability for various biomedical applications. Each type has distinct properties and applications depending on the nature of its polymer chains [33].

- *Homopolymeric* natural hydrogels are assembled using a single type of repeating unit derived from a natural source, obtained through extraction and purification processes, and are often biocompatible and biodegradable, forming gels through hydrogen bonding, for example, alginate or hyaluronic acid hydrogels used in wound dressings [34].
- *Copolymeric* natural hydrogels form by combining two or more different natural polymers through co-polymerization or physical blend. This combination allows physical, chemical and biological properties to be tuned to meet specific application needs, for example, gelatin-alginate hydrogels combine the cell adhesion properties of gelatin with the biocompatibility and gel-forming ability of alginate; chitosan-hyaluronic acid hydrogels combines the antimicrobial properties of chitosan with the water retention and biocompatibility of hyaluronic acid; collagen-chondroitin sulfate hydrogels mimic the natural extracellular matrix, helping to provide structural support and promote cartilage repair [35,36,37,38].
- *Hybrid* or *multipolymeric* natural hydrogels are composed of more than two different polymers. These hydrogels leverage the advantages of each component to create materials with superior performance and multifunctionality, formed by blending, cross-linking, or interpenetrating different polymers.

Alginate-chitosan-gelatin hydrogels integrate the gel-forming ability of alginate, the antimicrobial activity of chitosan, and the cell adhesion properties of gelatin. Hyaluronic acid-collagen-fibrin hydrogels combine the water retention of hyaluronic acid, structural support of collagen, and bioactivity of fibrin. Cellulose-hyaluronic acid-alginate hydrogels merge the mechanical strength of cellulose, the biocompatibility of hyaluronic acid, and the gel-forming ability of alginate.

Natural regenerative hydrogels thus offer a wide range of properties and functionalities. Homopolymeric hydrogels provide simplicity and consistency, copolymeric hydrogels allow for tailored properties, and hybrid hydrogels can mix the advantages of multiple polymers for enhanced performance in tissue regeneration and healing, drug delivery and other biomedical fields, leveraging their biocompatibility, biodegradability, and versatile properties [24].

Natural regenerative hydrogels can be designed to respond to various external stimuli: pH, temperature, chemical agents, or enzymatic activity, enabling controlled drug release or smart tissue engineering scaffolds [39].

*Based on their type of response*, they can be categorized as follows:

- *pH-responsive hydrogels*: undergo a significant modification in their swelling behavior or network structure in response to changes in pH, making them particularly useful for applications such as drug delivery, where the drug release can be activated by the pH differences in various parts of the body. Typically, pH-responsive hydrogels contain non-neutral groups that ionize or deionize affected by the surrounding pH; for example, chitosan contains amine groups that protonate in acidic conditions, causing swelling, allowing drug delivery systems targeting acidic environments like tumor tissues or the stomach, alginate contains carboxyl groups that ionize at higher pH, increasing swelling, applied in pH-sensitive oral drug delivery targeting the intestinal tract [40].
- *Temperature-responsive hydrogels* alter their swelling behavior or solubility as a reaction to temperature variations. They are usually made from polymers that undergo a phase transition at a given temperature and are useful for applications that require a gel-to-sol transition at specific temperatures, such as injectable drug delivery systems and cell encapsulation, for example, agarose or gelatin form gels upon cooling and melt upon heating (thermoreversible gelation), while methylcellulose forms gels upon heating above a certain temperature [41].
- *Chemical-responsive hydrogels* modify their properties as a consequence of specific chemical stimuli, such as ions or small molecules. This responsiveness allows for targeted and controlled drug release or other functional changes in the presence of certain chemicals. These hydrogels contain functional groups that interact with specific chemicals, leading to changes in swelling, degradation, or network structure; for example, alginate and pectin form gels in the presence of divalent cations like calcium ions, and chitosan swells or dissolves in the presence of certain anions or acidic conditions [42].
- *Enzymatic-responsive hydrogels* undergo degradation or structural changes in response to specific enzymes. This specificity makes them ideal for targeted drug delivery and tissue engineering applications where enzymatic activity is a trigger, leading to hydrogel degradation or structural changes upon enzymatic action; for example, collagenase degrades gelatin, hyaluronidase degrades hyaluronic acid, and chitosan is degraded by lysozyme [43].

Natural regenerative hydrogels exhibit a range of properties highly suitable for biomedical applications [44]. The main properties of natural regenerative hydrogels are as follows:

- *Biocompatibility*: the ability to interact with biological tissues without eliciting an adverse immune response. Ensures that the hydrogel can be safely used in medical applications without causing inflammation or rejection, like wound dressings and tissue scaffolds. Alginate, chitosan, and hyaluronic acid have excellent biocompatibility.
- *Biodegradability*: the capacity of the hydrogel to break down into non-toxic components that can be naturally absorbed or excreted. Essential for temporary scaffolds to avoid the need for surgical removal. Collagen and gelatin hydrogels degrade into amino acids that are easily processed by the body.
- *Swelling behavior*: the ability of hydrogels to absorb water and swell, which is influenced by the cross-linking density and the hydrophilicity of the polymer network, determines the hydrogel’s capacity to deliver drugs, absorb wound exudates and provide a moist environment for tissue regeneration. Alginate and hyaluronic acid hydrogels exhibit significant swelling.
- *Mechanical properties*: the strength and elasticity of the hydrogel are crucial for maintaining structural integrity under physiological conditions and critical in load-bearing scaffolds requiring mechanical stability for tissue engineering.
- *Gelation* and *rheological properties*: the process and behavior of hydrogel formation, including viscosity and flow under stress, influence the injectability and ease of application in minimally invasive procedures. Gelatin and agarose hydrogels exhibit thermoreversible gelation, useful for injectable applications where the gel forms in situ.
- *Bioactivity*: the ability of the hydrogel to interact with cells and biological molecules, promoting cellular adhesion, proliferation, and differentiation, essential to support cell growth and the formation of tissue. For example, collagen and fibrin hydrogels provide bioactive cues that assist cell attachment and growth, facilitating tissue regeneration.
- *Controlled release capabilities*: the ability to encapsulate and release therapeutic agents in a controlled mode is crucial to provide sustained and targeted release of drugs. Alginate and chitosan hydrogels can be engineered to release drugs in response to specific stimuli, such as pH or ionic concentration changes.
- *Stimuli-responsiveness*: the ability of hydrogels to respond to external stimuli such as pH, temperature, enzymes, or ionic strength allows for the development of smart materials that can adapt to the physiological environment for targeted and controlled therapeutic effects. For example, chitosan hydrogels are suitable for drug delivery in varying pH environments, such as the gastrointestinal tract.
- *Porosity*: the presence of interconnected pores within the hydrogel structure determines the diffusion of oxygen, nutrients and waste products, which is critical for cell survival and tissue integration. Hyaluronic acid hydrogels can be engineered with specific porosity to enhance cell infiltration and tissue regeneration.
- *Transparency*: The degree to which light can pass through the hydrogel is important for applications in ophthalmology and wound dressings where monitoring of underlying tissues is required. Hyaluronic acid and collagen hydrogels are often transparent, making them suitable for ocular applications and transparent wound dressings.

Natural polymers can be classified into several categories *based on their chemical structure*, including polysaccharides, proteins, and polyesters. Each class has distinct structural features and properties suitable for various biomedical and industrial applications.

Polysaccharides are polymers composed of chains of monosaccharide units linked by glycosidic bonds. They are highly hydrophilic and form hydrogels with excellent biocompatibility and biodegradability:

- Cellulose, the linear polymer of β-D-glucose units linked by β-1,4-glycosidic bonds, has high mechanical strength, is water-insoluble, suitable for biocompatible scaffolds and can be chemically modified to produce derivatives like carboxymethyl cellulose.
- Chitosan, the linear polymer of β-(1→4)-linked D-glucosamine and N-acetyl-D-glucosamine, is biodegradable, biocompatible, antimicrobial, and can form hydrogels in acidic conditions, suitable for use in wound dressings, drug delivery and antimicrobial coatings.
- Hyaluronic acid: the linear polymer of D-glucuronic acid and N-acetyl-D-glucosamine shows high water retention and promotes cell migration and proliferation. It is used in wound healing, drug delivery, and tissue engineering [45].

Polysaccharide gums are complex carbohydrates with high molar mass, soluble in water, which can form gels and mucilages. They are natural polymers derived from plants, seaweed or microbial sources, known for their hydrogel-forming ability and biodegradability, providing versatile and effective solutions for promoting tissue repair and regeneration. The most commonly used polysaccharide gums in regenerative medicine are as follows:

- Agarose, extracted from red algae (seaweed), is formed by alternating units of 3,6-anhydro-α-L-galactopyranose and β-D-galactose in a linear polymer. Agarose gels are stable at low concentrations, present thermoreversible gelation (melts at high temperatures and solidifies upon cooling) and have minimal immune response. It is used in cartilage scaffolds and neural tissue regeneration due to its supportive matrix for cell growth, as well as in controlled release systems for drugs and growth factors.
- Alginate, derived from brown seaweed, is the copolymer of β-D-mannuronic and α-L-guluronic acids, forms gels in the presence of divalent cations (Ca^2+^), is biodegradable and non-toxic and maintains a moist wound environment that promotes healing, suitable in scaffolds for cell encapsulation and tissue regeneration, particularly cartilage and bone and in encapsulation of drugs for sustained and controlled release [46].
- Carrageenan, extracted from red seaweed, is a sulfated polysaccharide with repeating units of 3,6-anhydro-D-galactose and D-galactose. It forms gels in the presence of K and Ca ions, is biodegradable, presents variable gel strength and texture depending on the type of carrageenan (κ, ι, λ), and is used in scaffolds for cartilage and in soft tissue regeneration.
- Gellan gum has a microbial origin (Sphingomonas elodea) and is a linear biopolymer formed by repeating units of glucose, rhamnose, and glucuronic acid. It forms strong, transparent gels in the presence of cations and is used in scaffolds for bone and cartilage tissue regeneration due to its ability to form stable gels that support tissue repair.
- Guar gum is a galactomannan composed of a linear chain of β-1,4-linked mannose units with α-1,6-linked galactose side chains, obtained from guar beans, presents high viscosity even at low concentrations and is used in wound dressings for its moisturizing and gel-forming properties.
- Xanthan gum, from the bacterium Xanthomonas campestris, is a polysaccharide with a backbone similar to cellulose (β-D-glucose) with mannose and glucuronic acid side chains, presents high viscosity and pseudoplastic behavior (shear-thinning properties), stable over a broad span of pH and temperatures. It is used in wound dressings for its gel-forming and protective properties.

Additional non-carbohydrate natural polymers of related interest are composed of amino acid monomers linked by peptide bonds. Due to the diversity of their amino acid sequences, they exhibit a broad diversity of physical and chemical properties.

- Collagen: a triple helix of three polypeptide chains forming a fibrous protein with high tensile strength, is biodegradable and promotes cell adhesion, is used in tissue engineering, wound healing, cosmetic surgery and medical implants [47].
- Gelatin: a denatured form of collagen consisting of single-stranded polypeptides, presents thermoreversible gelation, is biodegradable, and supports cell growth, is used in wound dressings and tissue engineering [48].
- Silk fibroin: composed mainly of glycine, alanine, and serine residues, with high mechanical strength, can be manufactured into various forms (fibers, films, gels) and used in tissue engineering, wound healing and drug delivery.

Each class of natural polymer offers unique properties that can be leveraged for specific biomedical applications, making them versatile materials for research and development in regenerative medicine, drug delivery, and tissue engineering. Polysaccharides are known for their hydrophilicity and gel-forming ability and proteins for their biodegradability and support for cell adhesion and growth.

Regenerative hydrogels based on natural carbohydrate polymers represent a promising frontier in biomedical engineering. Their inherent properties and versatility make them suitable for a broad span of applications, from wound healing to tissue engineering. Further research is peremptory to improve the functionality and applicability of regenerative hydrogels to ameliorate patient outcomes by different research strategies, combining natural polymers with other materials to strengthen hydrogels, developing hydrogels that respond to environmental changes (e.g., pH, temperature) for targeted therapy and creating hydrogels tailored to individual patient needs for more effective treatments.

## 3. Other Natural Materials Used in Obtaining Natural Hydrogels

The prevalence of infections induced by drug-resistant bacteria due to the abuse of antibiotics, the side effects, the possible complications, the high costs of the treatments, the instruments and the dedicated sanitary spaces and the specialized medical staff are just a few factors that underline the need for alternative approaches to the treatment of wound healing. As *innovative treatment strategies*, the use of hydrogels shows an increase in the topic of modern medicine. In addition to different usual approaches, including cellular immunotherapy, treatments by modifying gene expression, growth factor administration or skin grafts, the use of natural biomaterials, together with extracts of organic origin, vitamins, cells or bioactive agents, or bioinspired nanoparticles, are promising approaches and avant-garde [49,50].

In an experiment that we latterly reported, we employed three natural polysaccharides (Acacia, Xanthan, and Guar gum) to obtain, through green synthesis, ZnO nanoparticles with controlled morphology as antibacterial agents. These nanoparticles were embedded in matrices of natural chitosan hydrogels functionalized with propolis extract. The test results revealed functional characteristics of the three types of natural hydrogels, usable as multifunctional biocompatible and efficient delivery systems of natural pharmacological agents for biomedical applications [51].

**Figure 3:**
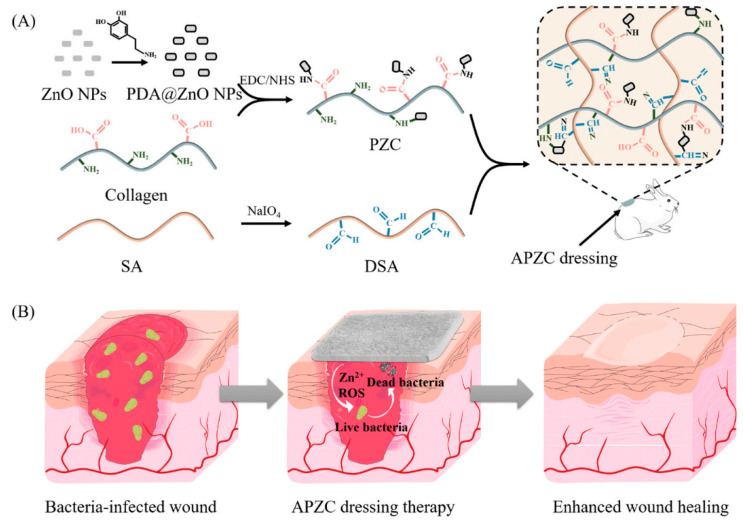
Schemes showing (**A**) the preparation of APZC dressings and (**B**) the antibacterial activity [52].

A well-targeted strategy is to obtain functional materials to combat a broad spectrum of bacteria designed to promote the healing of infected wounds throughout their surface and depth. New hydrogel nanocomposite dressings (APZC) were prepared based on collagen, incorporating ZnO in the form of a short rod coated with polydopamine (PDA) and cross-linked with dialdehyde sodium alginate. Biocompatible dressings with 3D network structure, tightly interconnected micropores and high physico-chemical properties had good blood coagulation capacity. In addition, they demonstrated effective antimicrobial activity that targeted a wide range of bacteria over a long period of time, reducing inflammation and leading to full-thickness healing of infected lesions without obvious scarring (Figure 3) [52].

Creatively developed, interactive wound dressings contain silver nanoparticles obtained through a green synthesis, reduced by folic acid (FA) through a metabolic activity and incorporated into a combinatorial platform of natural origin by -macromolecules of hybridized chitosan (CS) and collagen (COL). Along with good antimicrobial activity, the dressings demonstrated >98% wound healing assessed in hurtled rats and complete wound constriction to complete closure after two and three weeks, respectively [53].

Another method of preparing hydrogels was wet heat solution casting and fabrication of zinc oxide nanostructures in a ternary hydrogel model accommodating carboxymethyl cellulose-agarose-polyvinylpyrrolidone. The obtained biocompatible materials showed antibacterial activity, and an experimental in vivo wound healing research demonstrated the promotion of wound healing significantly within 18 days [54].

Inspired by the patterns and role of the extracellular matrix, a new multi-cross-linked hydrogel model containing hyaluronic acid, tyramine and chitosan glycol thiolate was reinforced with copper-doped bioglass nanoparticles. Experimental tests on full-thickness skin defects in mice revealed that sustained release of copper ions and silicon from the hydrogels induced migration of human umbilical vein-containing endothelial cells and accelerated healing, with complete repair of skin defects within two weeks [55].

Critical wounds developed over extensive surfaces, such as high-degree burns, are extremely demanding for both medical staff and patients due to the extreme pain, risk of infection, surgical procedures and long-term treatment protocols [56]. The different accidents or traumas that cause thermal, electrical or chemical burns can lead to large, damaged surfaces of different depths, sometimes deep, with oxidative stress, abundant exudation, and infectious risk, requiring effective materials such as covering dressings, skin transplantation or the employment some skin substitutes [57].

Designed as a dressing applicable in third-degree burn wound healing, a novel thermosensitive, in situ forming, double cross-linked hydrogel containing zwitterionic chitosan and starch dialdehyde was prepared for the delivery of silymarin and levofloxacin. Hydrogel injection on third-degree burns demonstrated remarkable antioxidant and antibiotic properties, with reduced inflammation and accelerated wound closure after 21 days [58].

## 4. Applications of Hydrogels Containing Natural Polymers for Wound Healing

Lesion healing is a natural but complex physiological evolution involving multiple, sometimes simultaneous, stages of hemostasis, inflammation, proliferation, remodeling, and maturation. In the hemostasis stage, there is an aggregation of platelets with the constitution of a provisional matrix of fibrin. Simultaneously, with the delivery of cytokines and growth factors in the wound by platelets, inflammatory cells, namely neutrophils, and macrophages, are activated. They activate fibroblasts and keratinocytes that induce the proliferative phase when ECM molecules such as collagen and proteoglycans are produced, and granulation tissue is formed, which replaces the provisional fibrin matrix.

Over time, the newly formed granulation tissue is covered by keratinocytes, and the wound closes. The growth factor of the vascular endothelium stimulates the endothelial cells, and the generation of fresh capillaries takes place, and type I collagen takes the place of type III collagen, maturing the granulation tissue [59].

Compared to normal wound healing, the biology of chronic is characterized by prolonged inflammatory, proliferative, or remodeling phases that can lead to tissue fibrosis or nonhealing ulcers [60].

### 4.1. Hydrogels Based on Proteins for Wound Treatment and Healing

The use of protein-based biomaterials, which are naturally large biomolecules and macromolecules, is often found due to valuable characteristics such as biocompatibility, biodegradability and favorable mechanical resistance, as well as an intrinsic natural biological activity [61,62].

Protein-based hydrogels have excellent mechanical properties [63,64] and are widely recognized as favorable materials that have demonstrated great potential for hemostasis, and the promotion of skin tissue healing and regeneration therapy [65,66]. Natural proteins, such as those of mussels, have adhesive capabilities and form underwater nanofibrillar systems by attaching to rocks underwater [67]. Various mixtures containing collagen, silk gelatin, fibrin or keratin are already commercially available as adhesive patches or injectable hydrogels or are new materials prepared and studied for use in wound healing, infection prophylaxis quick hemostasis, tailoring of adherence, or scar prevention and treatment [68,69].

This adhesive ability of mussels inspired a study in which a functional block was achieved by combining a mussel leg protein mimetic peptide with a mild amyloid central part sequence. The biocompatible hydrogel based on amyloid fibrils showed excellent mechanical and adhesion qualities, as well as an ability to improve cell proliferation. The hydrogel was improved with Ag nanoparticles due to the dense fibrillar structure with a considerable size of the specific surface, which allowed their incorporation. In vitro or in vivo studies showed that the release of AgNPs improved antimicrobial action by inducing membrane harm and reactive oxygen species (ROS) in bacteria and accelerated infected wound healing [70]. In another experiment, motivated by the remarkable wet adhesion capabilities of mussel proteins, natural hydrogels derived from plants were prepared. To make the hydrogels, soy protein isolates were used together with three selected polyphenols derived from plants, namely caffeic acid, chlorogenic acid and gallic acid. The obtained hydrogels showed biocompatibility, hemocompatibility, vapor permeability, good adhesion, excellent resistance under water and antibacterial activities suitable for wound treatment applications [71].

The collagen-based dressing materials obtained as hydrogels do not have good antibacterial activity or mechanical resistance. An injectable hydrogel designed as a wound dressing was made from collagen peptide functionalized with adipic acid dihydrazide, oxidized dextran, polyvinyl alcohol and borax. The self-healing ability of the codfish peptide-based hydrogel was assigned to numerous active reversible networks through the formation of acylhydrazone bonds, hydrogen bonds, imine bonds, and borate ester bonds. The antibacterial activity shown by the hydrogel was acceptable for both Gram-positive *S. aureus* and Gram-negative *E. coli* DH5α. The hemostasis and wound healing abilities of the hydrogel were evaluated in vivo in a rat hemorrhagic liver model and a rat burn model, respectively. The collagen-based hydrogel could be used as a wound dressing [72].

Following a wound, one of the first elements produced is the growth factor, which induces cellular responses in all stages of healing. Further, this activation improves cell migration and proliferation and enhances the production of the vascular endothelial growth motif and the insulin-like growth stimulator. As a result, there is an enhanced manifestation of the growth motif receptor and the production of extracellular matrix (fibronectin, hyaluronic acid) [73].

The complexity of the mechanisms of action required in the evolution of healing, growth factor dependence and active tissue regeneration has led to modern approaches and specific therapies as treatment modalities [74].

Topical use of growth motifs can contribute remarkably to wound healing, but the rapid reduction of growth factors over time requires specific delivery systems for the healing effects to be potent [75].

Numerous studies have investigated ways of advanced delivery of growth factors, knowing that after transplantation, their effectiveness is frequently attenuated due to fast degradation and non-targeted placement. A recent study harnessed the capacity of nano-fibrillated cellulose (NFC) hydrogel as a bio-compound carrier of platelet-rich plasma. The materials were investigated in a scratch wound model and diffusion studies in vitro, as well as in a full-thickness excisional wound model in SKH1 mice in vivo. The determinations revealed that the nano-fibrillated cellulose hydrogel is a suitable formulation for both accelerated wound healing and as a carrier for platelet-rich plasma with localized and controlled release in skin tissue engineering applications [76].

Hydrogels based on collagen with hyaluronan or sulfated hyaluronan content can contribute to wound healing by constantly releasing the epidermal growth factor-like factor that binds heparin [77]. The ability of hyaluronan, as well as its sulfated derivatives, to bind and release this growth factor from the obtained hydrogels was investigated by in vitro experiments. The results indicated a bioactivity of at least 72 h of the hydrogels, an induction of keratinocyte migration, and the highlighting of heparin-binding growth factor and HGF indicator in dermal fibroblasts. Also, in a porcine skin organ culture model, it was determined that hydrogels containing sulfated hyaluronan restored re-epithelialization in epithelial injury [78].

It is known that angiogenesis is an important physiological stage in tissue healing. In a study for the control of anti-inflammatory and angiogenic action for accelerating tissue healing, gelatin-based acellular heparinized hydrogels, sensitive to magnetic field, seeded with mesenchymal stem/stromal cells and magnetic nanoparticles were prepared. Test results suggested that heparinized acellular hydrogels efficiently retain angiogenic growth factors released by magnetically activated mesenchymal stem/stromal cells, promoting angiogenesis and improved tissue regeneration [79].

### 4.2. Hydrogels Based on Polysaccharides for Wound Treatment and Healing

Polysaccharide-based hydrogels are innovative, natural, and advantageous drug delivery systems and are widely applied in wound management due to their bioavailability, stability, solubility, and targeted delivery of phytocomponents [80,81]. Polysaccharides can be obtained from natural sources, such as plants, bacteria, algae and animals, and are abundant natural biocompatible, biodegradable, hydrophilic polymers that contain functional groups suitable for encapsulating active biocomponents [82].

The versatility of polysaccharide-based hydrogels consists of the following:

(i) Compatibility with biological systems;
(ii) Their possibility of encapsulating some phytocomponents with intrinsic biological activity;
(iii) Their controlled release capacity with many benefits for human health [83,84].

In addition, characteristics similar to biological tissues make them increasingly attractive for medical and pharmaceutical applications [85,86,87,88]. They can manifest as a scaffold support for various living cells, being able to restore their microenvironment to a normal physiological and pathological state. This aptitude to mimic the extracellular matrix of a particular organ or tissue allows hydrogels to be used as biological tissue analogs for application in both tissue-engineered constructs and 3D in vitro models [89].

Some other advantages of polysaccharide-based hydrogels include short gelation time, the possibility of not using cross-linking agents, ease of topical application and injectability [90]. Due to the multitude of advantages of hydrogels containing polysaccharide, their application in regenerative medicine is highlighted by a huge number of valuable studies developed, as well as by the number of effective materials manufactured on this topic [91].

The technology of wound healing dressings has advanced tremendously in recent times with added elements that improve specific characteristics and functionalities such as reactivity to external factors, adhesiveness, mechanical robustness regulation, the release of therapeutic factors, antioxidant and antimicrobial properties, and a new way of stimulating tissue regeneration.

Composite hydrogels based on natural polysaccharides are excellent topical wound dressings. They can be designed to have therapeutic effects on multiple types of wounds depending on various parameters such as model surface area and depth of the wound, exudate levels, and mode of application for patient comfort. It supports the incorporation of additional elements with synergistic effects as special healing accelerator agents [92,93].

Such a composite dressing was obtained from natural polysaccharides, namely gellan gum and alginate. The mixture that also included lipid nanoparticles with antibacterial peptide content—nisin was designed for the treatment of infected wounds. The prepared materials showed cytocompatibility and functional antimicrobial activity toward Gram-positive Streptococcus pyogenes, with a promising potential for applications as dressings for the healing and treatment of bacterial wound infections [94].

Hydrogels based on selectively oxidized polysaccharides have been used as the basis for the development of a series of hemostatic wound dressings in the form of advantageous cryogels. The structures were designed selectively, combining hemiacetal groups, dialdehyde pullulan, and dopamine. The prepared materials showed good mechanical stability and hemostatic activity and were proposed as potential dressings for wound management [95].

The worldwide increase in the cost of skin wound treatments has led to a rapid evolution of sustainable material science with predetermined properties, especially for the development of new effective, bioavailable and low-cost dressing products.

Natural gels, for example, Aloe vera, honey or other bee products, have a synergistic effect, being used both as a native therapeutic tool and as a supplier for other embedded bioactive elements. The Aloe vera plant has been used for thousands of years by many civilizations for its medicinal and nutritional properties in skincare or beauty. Recently, hydrogels containing Aloe vera have been developed as potential low-cost platforms for the delivery of healing factors in active dressings for wound healing due to their biocompatibility and unique properties of action and biological activity. Aloe vera gel contains a multitude of bioactive phytocompounds such as polysaccharides (approx. 55%), sugars, minerals, proteins, lipids and phenolic compounds [96,97,98].

**Figure 4:**
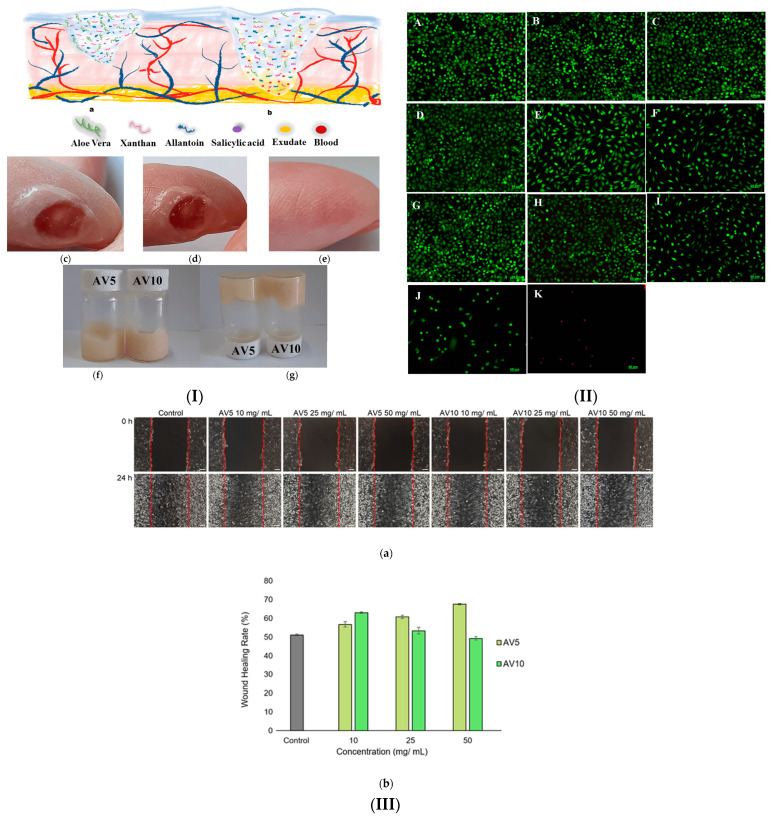
(**I**) Aloe vera-based dressing: (**a**) dry and (**b**) wet hydrogel structure. Skin wound with time (**c**) 0 min; (**d**) 5 min; (**e**) 20 days; (**f**,**g**) images showing inverted vials with AV hydrogels. (**II**) Fluorescent images for dead and live L929 cells (**A**) control; (**B**–**F**) AV5 treated; (**G**–**K**) AV10 treated (48 h). Hydrogel concentrations are (**B**,**G**) 10 mg/mL; (**C**,**H**) 25 mg/mL; (**D**,**I**) 50 mg/mL; (**E**,**J**) 75 mg/mL; (**F**,**K**)100 mg/mL. (**III**) Healing after wound generation in vitro (24 h): (**a**) optical micrographs. (**b**) closure percentage [106].

Numerous studies have used and investigated the multiple functions, biological properties and pharmacological effects for various uses, knowing the positive impact proven for centuries [99,100,101]. Various natural hydrogel dressings have been developed using AV as an active ingredient [102,103,104,105]. In two recent studies we have designed biocompatible natural hydrogels based on Aloe vera obtained through an ecological synthesis method for the treatment and healing of skin lesions (Figure 4) [106,107].

Along with other components of natural origin, such as salicylic acid, allantoin and xanthan gum, we brought Aloe vera in different concentrations (5–20%) [107]. When applying hydrogels to a wound, preliminary tests indicated rapid absorption in the wound area, a feeling of pain relief and good hydration. The studies on the physico-chemical, pharmaco-technical and rheological characteristics of the prepared hydrogels indicated a good viscoelastic behavioral profile and favorable stable and specific characteristics of cross-linked structures. The antibacterial activity evaluated on Gram-positive strains of Staphylococcus aureus and on Gram-negative strains of Pseudomonas aeruginosa demonstrated that the hydrogels have very good antibacterial capacity (Figure 4). Also, the in vitro scratch test proved the capability of hydrogels to accelerate cell proliferation and migration and to induce closure of a wounded area. The hydrogels obtained based on the natural components of the Aloe vera gel could be developed as bioproducts in wound healing applications.

An interesting and useful study on the release of drugs from polysaccharide-based hydrogels was carried out both experimentally and theoretically on cyclodextrin-k hydrogels cross-linked with carrageenan. The hydrogel films based on beta cyclodextrin and kappa carrageenan were obtained by cross-linking with epichlorohydrin and characterized physico-chemically, then a new theoretical model was proposed for the release of the drug from the films [108].

Refractory wounds continue to require and cause various advanced studies for the development of new products based on natural materials, due to the enormous suffering caused to patients. Among the recently developed approaches in regenerative medicine, stem cell therapy represents a revolution in regenerative therapies. Various stem cells have been investigated for the treatment of numerous degenerative disorders, functional defects, and wound healing, including bone marrow-derived stem cells, human dermal fibroblasts, amniotic membrane, umbilical cord (UC), adipose-derived stem cells (ASCs), fetal tissues, circulating angiogenic cells, and keratinocytes [109].

Adipose tissue is an accessible, abundant and attractive source because mesenchymal stem cells (purity 95%) useful in healing skin lesions can be obtained with high yield. Stem cells obtained from adipose tissue have proliferative capacities to secrete paracrine factors that initiate the work of tissue regeneration. The mechanism of action, the ability to secrete pro-angiogenic growth factors, and their safety profile give them the advantage of being used as perfect materials in the therapy of refractory wounds [110]. In an in vivo study, the impact of Aloe vera hydrogel containing allogeneic adipose stem cells using a rat burn wound template was evaluated. Different hydrogels of Aloe vera 50% and ASC, Aloe vera and demineralized bone matrix (DBM), as well as DBM-Aloe vera/ASC composites, were transferred to the wound area by intradermal injection. The highlighted effects showed the ability of the DBM-Aloe vera/ASC composite hydrogel to reduce inflammatory responses and to determine the generation of much fewer scars compared to the other two types of hydrogels, being able to stimulate the healing of burn wounds [111].

By using the solvent casting method containing curcumin, an injectable hydrogel based on chondroitin sulfate and sodium alginate, self-assembled and cytocompatible, was obtained. It exhibited favorable tissue regeneration capacity and showed improved wound healing in contrast to control groups. Curcumin’s pharmacokinetic (PK) profile and in vivo toxicity were assessed by subcutaneous injection over 24 h during controlled release studies. The results confirmed the ability of injectable hydrogel for accelerated healing of diabetic wounds [112].

Treating hard-to-heal wounds is a continuous challenge for the scientific and medical world. Diabetic foot ulcers are devastating complications due to hyperglycemic states, which require long-term care and treatment programs to aid wound healing. Foot ulcer dressings are a key component of the healing process, with many different types currently available, including hydrogel dressings. A hydrogel-based diabetic wound patch was prepared from natural origin polymers such as chitosan and gelatin with polyvinyl pyrrolidone and reinforced with cellulose nanofibrils to increase its stability. The hydrogel was impregnated with a nanoemulsion of oregano essential oil, and low-level laser therapy was used. In vivo test results on diabetic foot ulcer rat models showed rapid healing effects, with a maximum healing rate of 97.5%. Combined nanoemulgel therapy with low-level laser resulted in minimal scarring formation. On the other hand, a significant decrease in inflammation was observed during treatment due to an increase in granulation and improved re-epithelialization [113].

Wounds of combined radiation and burn injuries are serious wounds that require special management and complex long-term treatment and are due to hematopoietic, immunological suppression and reduction of stem cells, which are associated with redundant reactive oxygen species (ROS). For this purpose, to accelerate the healing of wounds of this type by scavenging ROS, an injectable multifunctional Schiff base was made by designing it and cross-linking it with gallic acid-modified chitosan (CSGA)/oxidized dextran (ODex) hydrogels. The obtained biocompatible and easily injectable hydrogels showed strong antibacterial and antioxidant activity and accelerated wound healing in mice with combined radiation and burn injuries, showing potential as dressings for the clinical treatment of severe wounds [114].

Recently, certain bacteria that have the ability to cause severe illness are becoming resistant to frequently used antibiotics, and the infections they cause require expensive, long-term treatments and involve specialized medical personnel. Antimicrobial-resistant bacteria include methicillin-resistant Staphylococcus aureus (MRSA), vancomycin-resistant Enterococcus (VRE), multi-drug-resistant Mycobacterium tuberculosis (MDR-TB) or carbapenemase-producing Enterobacterales (CPE).

New materials dedicated to the inhibition and eradication of drug-resistant bacteria have been designed using natural polysaccharide materials with special biological characteristics. By combining quaternized insect chitosan-catechol, pullulan methacrylate-dialdehyde and gallium ion, a photo-enhanced multi-cross-linked hydrogel was made. Test results revealed the promotion of wound reduction, collagen deposition and angiogenesis, conducting to the elimination of inflammation in a whole-skin wound model of MRSA-infected rats [115]. Table 2 presents a comparative analysis of polysaccharide-based hydrogels used in the treatment and healing of injuries presented in this sub-chapter.

### 4.3. Hydrogels Based on Glycosaminoglycans for Wound Treatment and Healing

Glycosaminoglycans (GAG) are ancestral molecules with a fundamental role in the management of vital biological processes, regulating cell proliferation and differentiation, matrix construction, homeostasis and cell behavior [121]. With a complex and diverse structure, glycosaminoglycans are ubiquitous in cells and vital to biological systems, but more elements have yet to be discovered concerning their specific functions [122].

Among important classes of GAGs, the most important include hyaluronan (HA), chondroitin sulfate (CS), keratan sulfate (KS), and heparin sulfate (HS). GAG-based hydrogels are used in various cell and tissue culture applications. By modulating cytokines administration, these hydrogels demonstrated good functional activity as a result of their design as in situ assembly materials. This focused design helped develop a hydrogel platform with variable sulfation patterns of bio-orthogonal cross-linked star-shaped GAGs and efficient control of embedded mesenchymal stem cells (MSCs) as suitable in situ assembly materials for cell embed-ding and tissue injection. These hydrogel platforms could be developed for advanced 3D cultures and precision tissue engineering [123].

**Figure 5:**
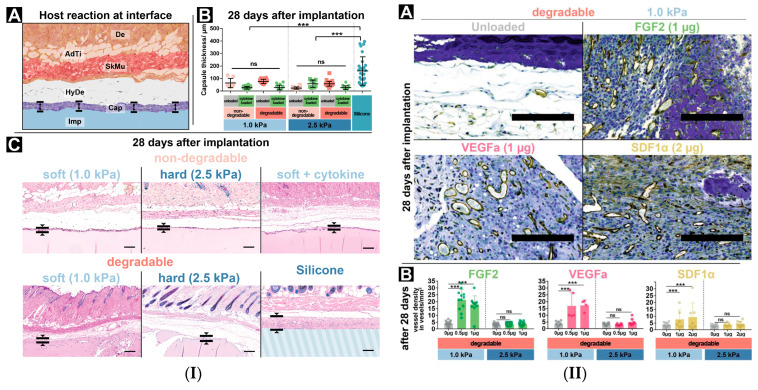
(**I**) PEG-GAG hydrogels implanted subdermally. (**A**) Scheme of the implantation site: Dermis (De), Adipose Tissue (AdTi), Skeletal muscle (SkMu), Hypodermis (HyDe), Capsule (Cap), Implant (Imp). (**B**) Foreign body reaction thickness, ns—not significant, ***—*p* ≤ 0.001. (**C**) Immunostaining after 28 days. Scale bar 200 μm. (**II**) Angiogenic effects, hydrogels loaded with different cytokines. (**A**) CD31 immunostaining of hydrogels (dark blue) with vascular structures (brown). Scale bar 200 μm. (**B**) Vessel formation [125], ns—not significant, ***—*p* ≤ 0.001.

Another study focused on hydrogel matrices based on silk proteins (Antheraea mylitta), sericin, chitosan (for antibacterial and structural support) and glycosaminoglycans (a component of biologically formed extracellular matrix), incorporating vascular endothelial growth factor and transforming growth factor -β have been investigated for skin repair in vitro and in vivo [124]. In vivo studies evidenced that biocompatible porous hydrogels mimicking natural skin tissue, functionalized with dual growth factor promoted skin tissue repair, angiogenesis and minimally illicit immune response [124]. One in vivo study evaluated the host feedback to in situ assembled PEG-GAG hydrogels by subcutaneous implantation in immunocompetent C57BL/6J mice for up to 28 days. The immune reaction and the angiogenic response to the GAG-based hydrogels, with systematically varied cytokine functionalization, as well as other physical characteristics of cellular adhesiveness and enzymatic degradability, were highlighted. Their results showed that by implanting GAG-based hydrogels, tissue reactions to the foreign body can be managed in an effective way by adapting the hydrogel system in situ, the materials being safe for tissue engineering applications in vivo (Figure 5) [125].

**Figure 6:**
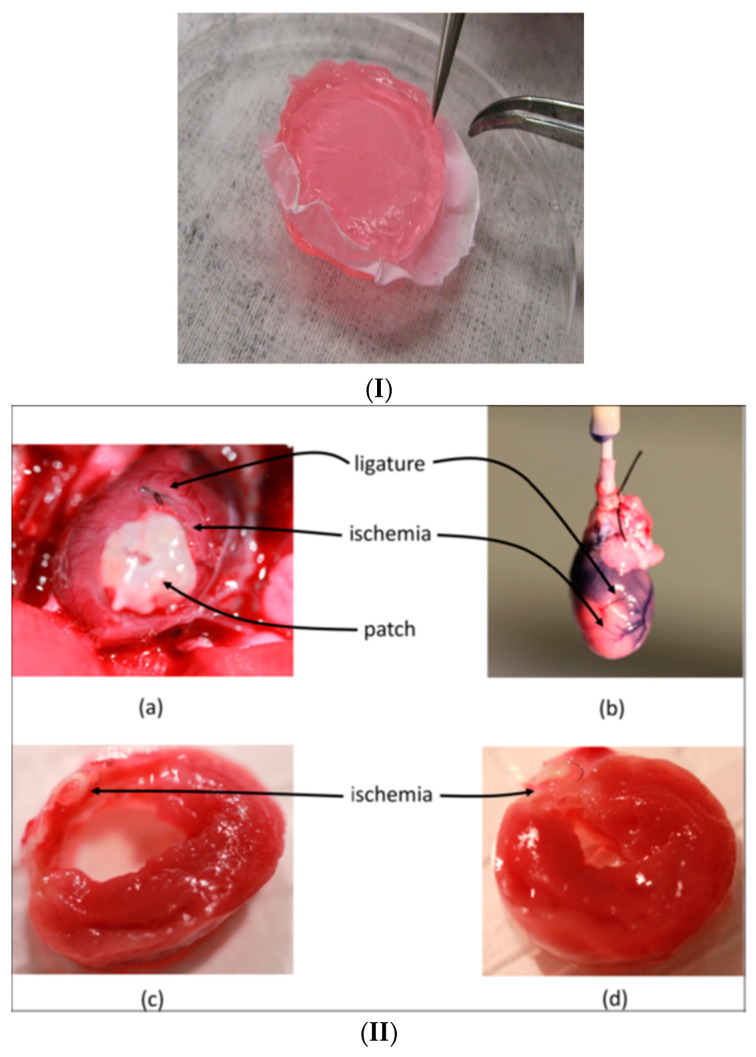
(**I**) Optical image of the reinforced hydrogel. (**II**) (**a**) Image of the heart before explantation showing the white patch, the pink ischemic region and the coronary artery ligature. (**b**) Image of the heart after explantation, showing the ischemic region. Images of the transversal section through the ventricle wall, post-implantation, for (**c**) the control case without growth factors, and (**d**) case treated with HA:Hp-silk patch containing growth factors, showing a reduced damaged region, nearly full wall thickness, and significantly less fibrous deposition [126].

An interesting and particularly extensive project was dedicated to obtaining beneficial therapeutic responses for diseased hearts by co-delivery of growth factors, which have the potential to promote revascularization and restoration of the ultrastructure of tissue in the ischemic border region. An assessment was conducted in vivo on the capacity showed by composite hydrogels that include hyaluronic acid and heparin preloaded with both vascular endothelial growth factor (VEGF) and platelet-derived growth factor (PDGF) and reinforced with silk by measuring the generated histological responses. The prepared composite hydrogels were applied to Sprague-Dawley rats by sutures as patches on the ventricular surface of the ischemic myocardium. The angiogenic response recordings were monitored for 28 days. Favorable responses were recorded for the hydrogels in ischemic myocardium due to the growth factors delivered, which decreased both cell apoptosis and the density of challenged mast cells and CD68+ and anti-inflammatory CD163+ macrophages (Figure 6) [126].

**Figure 7:**
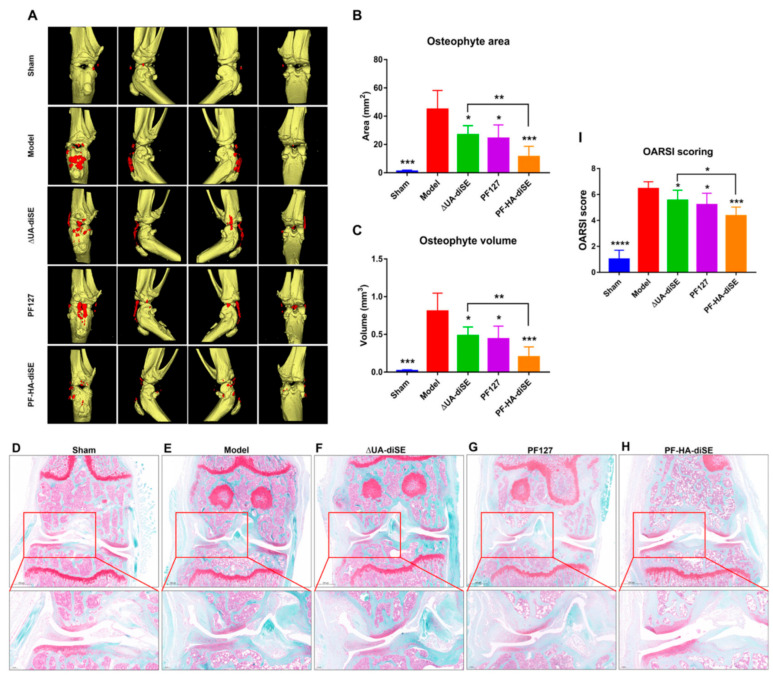
(**A**) Computed tomography 3D images of knee joints treated 50 days with hydrogels, area (**B**) and volume (**C**) of osteophytes. * *p* < 0.05, ** *p* < 0.01, *** *p* < 0.001, n = 5. (**D**–**H**) Safranin staining of the paraffin sections of knee joints. An enlarged view is shown at the bottom of each image. (**I**) OARSI score of sections stained with safranin O-fast green. * *p* < 0.05, *** *p* < 0.001 and **** *p* < 0.0001, n = 5 [127].

Osteoarthritis (OA) is a chronic, degenerative joint condition that involves cartilage and many of the surrounding tissues. Slowly progressing and causing great pain, with joint failure up to disability, OA affects hundreds of millions of people worldwide. The prevalence of the disease is increasing, with huge costs, because the treatment to reduce the progression of the disease is not yet accessible, and the manufacture of prostheses and elaborate surgical interventions to replace the joints are necessary. Over time, numerous materials dedicated to this disease have been developed and investigated. Among them, in an experimental study, an injectable hydrogel based on glycosaminoglycan was developed, containing hyaluronic acid (HA), chondroitin sulfate E disaccharide (ΔUA-diSE) and thermosensitive Pluronic F127 (PF127). It was investigated both in vitro and in vivo to test the slow-release capacity, associated mechanisms and disease efficacy of PF-HA-diSE (Figure 7). The results, which indicated the formation at body temperature of the thermosensitive hydrogel and the maintenance of a slow and sustained release of ΔUA-diSE, with joint lubrication and anti-inflammatory effect, are encouraging for clinical applications (Figure 7) [127].

Another recent study was conducted for a progressive shoulder condition that causes stiffness, inflammation, and fibrosis of the glenohumeral joint, known as frozen shoulder (FS). The thermosensitive hydrogel designed to be used in injectable form was prepared based on hyaluronic acid/Pluronic F-127 (HP), loaded with dexamethasone and collagenase (HPDC). After conducting drug release tests in both vitro and vivo for approximately 10 days, it was found that inflammation was inhibited, and excess collaged fibers were degraded [128].

As wound healing involves multiple factors, such as variations in patients’ or donors’ age, associated diseases, the anatomical area or the level of development of the organ, there is no specific predetermined model of treatment. Current clinical therapeutic approaches involve various innovative therapeutic strategies to obtain effective bioactive materials that influence and lead the healing process in the desired direction.

A new modern strategy that addresses cellular behavior is the improvement of the functionality of stem cell exosomes in accelerating the healing of skin wounds. Recombinant human collagen I and carboxymethyl chitosan were used to create hrCol I/CMC-Exos, a hydrogel that contains exosomes extracted from human umbilical cord mesenchymal stem cells (hUCMSCs). The evaluation revealed its biocompatibility in vitro cell growth stimulation and maintained exosome release. The hydrogel effectively promoted the closure and regeneration of severe skin wounds in the mouse complete skin defect model through a mechanism of increasing fibroblast proliferation, decreasing inflammation and promoting angiogenesis [129].

## 5. Natural Hydrogels as Bioinks for 3D Bioprinting

**Figure 8:**
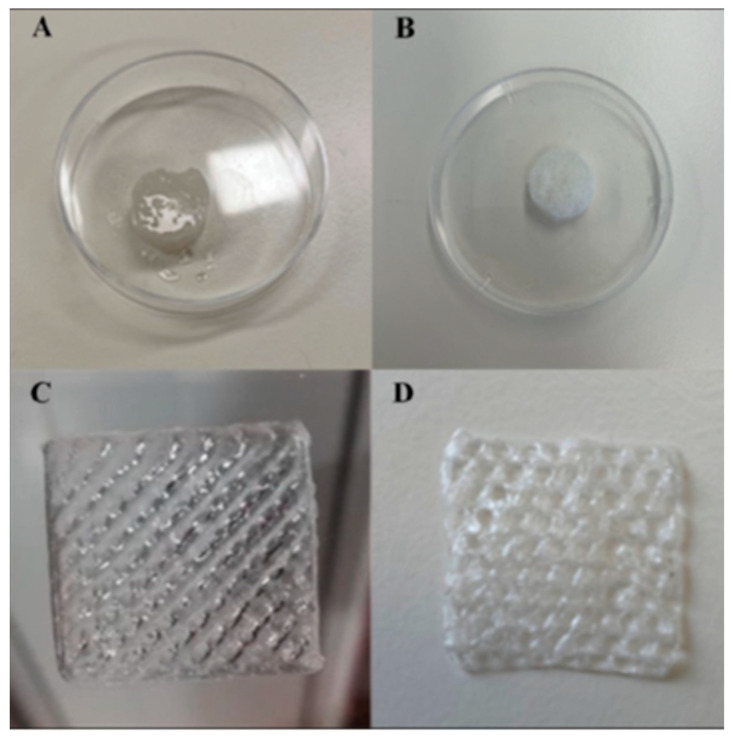
Hydrogel scaffold: (**A**) before; (**B**) after lyophilization. Three-dimensional printed scaffold: (**C**) before; (**D**) after lyophilization [135].

There is growing interest in the introduction of natural hydrogels in smart formulations as bioinks for three-dimensional (3D) printing [130]. Bioinks facilitate producing complex structures such as natural hydrogels, cell aggregates, bioactive compounds, acellular matrix, etc. [131]. These bioinks require hydrogel formulas with good physico-chemical and biological characteristics and with lower viscosity to be able to pass through the printing needle and be processed by automatic bioproduction technologies [132,133]. The clinical applications of 3D bioprinting are relevant for wound healing and regenerative medicine, for the regeneration of various tissues (skin, nerves, muscles, bones, heart, and cartilage) [134]. A new bio-ink was designed for therapeutic purposes by making a 3D printed hydrogel to be implanted locally in the infarcted cardiac tissue. A polymeric matrix was made from two natural polysaccharides (Gellan Gum and Konjac glucomannan) containing human umbilical vein endothelial cells (HUVEC) as angiogenic factors. The result was a bioactive hydrogel characterized by stability, biocompatibility, bioactivity and a porous structure. Printing led to obtaining a 3D structure realized at low costs with the potential of use in cardiac tissue regeneration (Figure 8) [135].

A mixture of methacrylate gelatin, cells, type I collagen and a photoinitiator were cross-linked with UV light to obtain hydrogels as 3D printable biological inks. Following the printing process, distinctive capillary-like structures were obtained. By adding collagen, a formulation was created with improved biological and rheological properties and adequate for 3D bioprinting of pre-vascularized while promoting angiogenesis [136].

Advanced studies using the photo-cross-linking method were developed due to the need to obtain a scaffold with high cell viability and structural stiffness relevant for chondral repair. The core component was formed from natural methacryloyl gelatin/hyaluronic acid methacrylate hydrogel incorporating mesenchymal stem/stromal cells derived from infrapatellar fat, while the same hydrogel (GelMa/HAMa) became the outer component (shell) of a coaxial system and was photo polymerized. The obtained bio-ink made it possible to create GelMa/HAMa bio-scaffolds in the form of core-shell structures and almost 200 kPa stiffness after 10 s of UV-A exposure (365 nm, 700 mW/cm^2^). Surprisingly, under these conditions, the bio-scaffolds managed to keep more than 90% of the stem cells viable, which kept the ability to proliferate, thus showing a great potential for in situ surgical engineering of cartilage [137].

**Figure 9:**
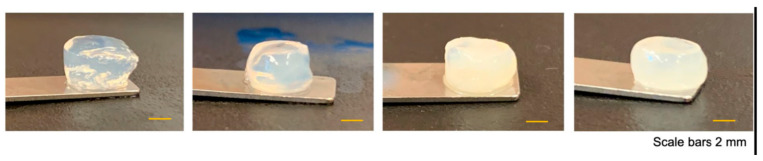
Image of hydrogels combining silk fibroin and tyramine-substituted gelatin with the human placental extracellular matrix, showing changes in transparency according to composition [138].

**Figure 10:**
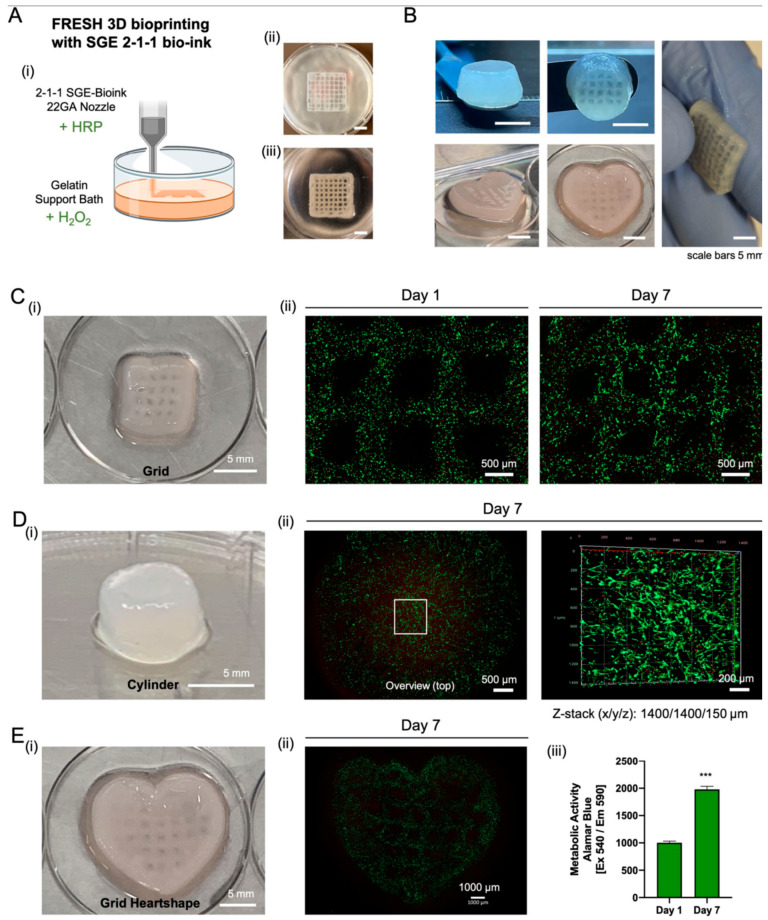
Three-dimensional printed SGE hydrogel containing human fibroblasts. (**A**): (**i**) scheme of the 3D printing process showing the combination of cross-linking agents in the support bath. (**ii**) Scaffold printed into the support bath and (**iii**) after washing. (**B**): Different shapes 3D printed with SGE hydrogel. (**C**–**E**) (**i**): printed scaffolds. (**C**–**E**) (**ii**): staining of live and dead cells after one and seven days. (**E**) (**iii**): Metabolic activity increases over time (*p* < 0.05, Alamar Blue staining, *** indicates significant differences of *p*  <  0.05 between Day 7 and Day 1) [138].

In a very recent study, a new innovative natural hydrogel was designed to simulate natural tissue biomechanical properties through enzymatic cross-linking of silk fibroin and tyramine-substituted gelatin with the human placental extracellular matrix. This new composite hydrogel formulation was then applied in optimized (3D) bioprinting to produce biocompatible artificial soft tissue with biomechanics that mimic natural tissue (Figure 9) [138]. According to in vivo test results, the implanted material was shown to have responded favorably, as they displayed biocompatibility, bioactivity and stability after 90 days, good with suitable biomechanical properties and no necrosis over time (Figure 10) [138].

**Figure 11:**
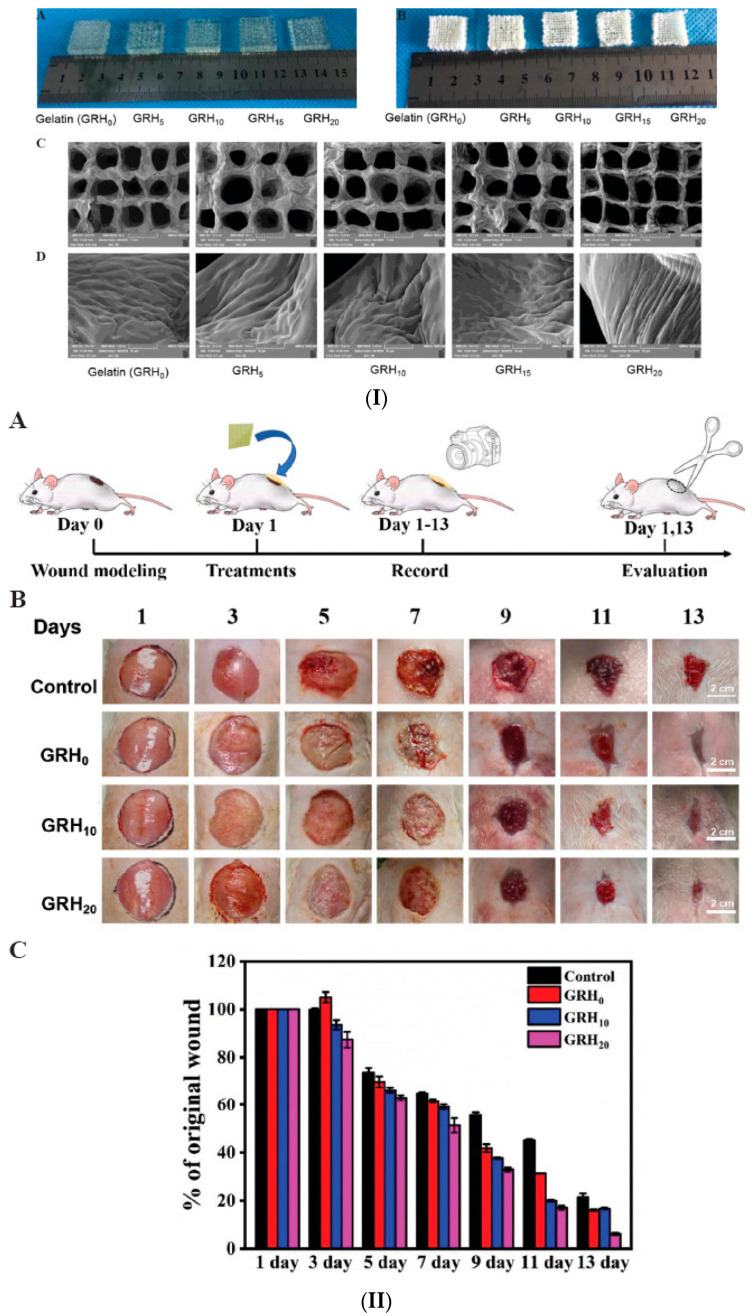
(**I**) Optical micrographs of GRH gelatin-based hydrogel scaffolds: (**A**) wet; (**B**) lyophilized and SEM micrographs (**C**) 50×; (**D**) 1000×. (**II**) (**A**) Scheme of wound evolution evaluation. (**B**) Optical images of wounds treated with GRH for 13 days. Scale bar: 2 cm. (**C**) Reduction of the wound sizes (% of the initial wound size, n = 6) [139].

In clinical practice, it is known that artificial skin can save patients with severe burns or on large surfaces from the painful intervention of autologous skin transplantation. The continuous search for effective transplant substitutes for clinical use has led to the reporting of numerous studies dedicated to biocompatible and printable biomaterials, which have the potential to facilitate effective skin regeneration. Such a material was prepared in the form of a hydrogel by enzymatic cross-linking, based on gelatin and collagen hydrolyzate, from recombinant human collagen type III (rColIII). The 3D-printed hydrogel was tested as a dressing and showed therapeutic efficacy in a rat skin lesion model (Figure 11) [139]. Table 3 illustrates the key features of recent experiments on the biological functions and applications of polysaccharide-based hydrogels.

## 6. Challenges, Opportunities and Possible Perspectives

The application of natural regenerative hydrogel polymers in wound healing is a promising area of research with the potential to revolutionize medical treatments. These hydrogels offer unique properties that make them suitable for promoting tissue regeneration and accelerating the healing process. Natural regenerative hydrogels can mimic the extracellular matrix, providing an ideal scaffold for cell proliferation and tissue regeneration. Moreover, utilizing hydrogels as delivery vehicles for bioactive molecules, such as growth factors, cytokines and antibiotics, can further enhance their healing properties. Advances in 3D printing and biofabrication techniques allow for the customization of hydrogel shapes and properties to specific wounds. Hydrogels are particularly beneficial for chronic wounds like diabetic ulcers and pressure sores. However, like any emerging technology, they come with their own set of challenges. Here, we try to briefly overview the most significant challenges and foreseen possible perspectives associated with their use in wound healing:

- *Biocompatibility and toxicity*: Ensuring that hydrogels are biocompatible and do not induce immune responses or toxicity is crucial. Some hydrogels may degrade into harmful by-products. Rigorous testing and modification of the hydrogel composition to eliminate any toxic components can solve this challenge. The use of completely natural polymers, like alginate, chitosan, and collagen, which are inherently biocompatible, can certainly mitigate such risks.
- *Mechanical properties*: Hydrogels often have poor mechanical strength, which can limit their application in areas that experience stress or movement. Reinforcing hydrogels, either with cross-linking agents or by incorporating a reinforcing agent such as nanoparticles/nanofibers to enhance mechanical strength, can solve this issue.
- *Controlled Degradation Rate*: The rate of hydrogel degradation needs to match the rate of tissue regeneration, which can be difficult to control. Developing hydrogels with tunable degradation rates through the use of smart polymers or hybrid systems can ensure that the material supports the healing process effectively.
- *Moisture Retention*: Maintaining the right moisture balance is critical for wound healing. Excess moisture can lead to maceration, while insufficient moisture can slow down healing.
- *Cost and Scalability*: Production costs and scalability can be significant barriers, especially for high-purity natural polymers. The use of readily available natural resources can reduce costs and improve scalability, being a tremendous opportunity for the rapid development of affordable regenerative hydrogel technologies accessible to low- and middle-income patients.

Collaboration between material scientists, biologists, and medical professionals is essential to drive innovation and improve hydrogel formulations for wound healing. *Interdisciplinary research* centers and partnerships are necessary to foster the exchange of ideas and accelerate the development of new technologies. The perspective of integrating hydrogels with emerging technologies such as biosensors or wearable devices can create smart wound dressings that monitor healing progress and adjust treatment in real-time.

Future fundamental research should focus on understanding the complex interactions between hydrogels and biological systems to optimize their performance. From the interaction of the natural components of the hydrogels and the target cells or pathologies in complex physiological circumstances, a biological response is obtained that must be conducted correctly and improved in future studies. Accurately interpreting and regulating this specific response is crucial and can determine both the time and quality of the wound-healing process.

The clinical use of these biomaterials still requires improvements in many aspects, such as preparation and standardization. Investing in clinical trials will provide insights into the mechanisms of action and help refine hydrogel technologies.

Finally, emphasizing the use of sustainable and renewable natural resources in hydrogel production aligns with environmental goals and reduces ecological impact. Research into biodegradable polymers and waste-reducing manufacturing processes can promote sustainability.

## 7. Conclusions

Natural carbohydrate-based hydrogels represent a versatile and promising class of materials in regenerative medicine and tissue engineering. Their intrinsic biocompatibility, biodegradability and structural similarity to the extracellular matrix make them ideal candidates for a wide range of biomedical applications. This review has highlighted the significance of key carbohydrate polymers, including alginate, chitosan, and hyaluronic acid, in developing hydrogels with tailored mechanical and biological properties. Advances in cross-linking methods, both physical and chemical, have further enhanced the functionality and applicability of these hydrogels in drug delivery, wound healing, and scaffold construction for tissue regeneration. Despite significant progress, challenges such as precise control over degradation rates, mechanical strength, and scalability remain. Future research should focus on optimizing these properties and exploring novel natural sources, hydrogel formulations and cross-linking strategies to improve the performance and clinical translation of these hydrogels. Overall, the continued development of regenerative hydrogels based on natural polymers holds great potential for advancing healthcare and improving patient outcomes in various regenerative therapies.

## Figures and Tables

**Table 1:** Comparison of physical or chemical cross-linking methods.

Feature	Physical	Chemical
Bond Type	Non-covalent interactions	Covalent bonds
Reversibility	Generally reversible	Generally irreversible
Conditions	Mild, often physiological conditions	May require specific reagents or conditions
Biocompatibility	High, no need for toxic chemicals	Dependent on cross-linker and reaction by-products
Mechanical Properties	Often weaker, less stable	Stronger, more stable
Control over Properties	Limited	High, precise control over network structure
Applications	Wound dressings, cell encapsulation	Tissue engineering, drug delivery, durable scaffolds

**Table 2:** Polysaccharide-based hydrogels applied in wound healing.

Biopolymer(s)	Compositional Attributes	Study	Results/Effects	Reference
Chitosan and collagen	Injectable thermosensitive hydrogels containing three-dimensional mesenchymal stem cell.	In vivo	Proliferation and secretion of paracrine effects, improvement of vascularization and re-epithelialization of the wound.	[116]
Chitosan and pectin	Thermoreversible biopolymer hydrogel.	In vitro	Excellent exudate absorption.	[117]
Sodium alginate and gelatin	Double network hydrogel incorporating self-assembled magnesium nanoparticles mediated by tea polyphenols.	In vivo	Healing of diabetic foot wounds infected with methicillin-resistant Staphylococcus aureus.	[118]
Sodium alginate	Double network hydrogel constructed with dopamine grafted oxidized sodium alginate and polyacrylamide and made by hydrogen bonding and dynamic Schiff cross-linking.	In vitro and in vivo	Self-healing and tissue adhesive hydrogel for wound dressing applications.	[119]
Collagen and starch	Injectable adhesive hydrogel based on starch modified with aldehyde incorporating collagen modified with dopamine.	In vivo	Injectable bioadhesive hydrogel with durable wet tissue adhesion, superior sealing performance, fast self-healing power, shape adaptability, and hemostatic properties.	[120]
Gellan gum and sodium alginate	Composite dressing based on natural polysaccharides and lipid nanoparticles containing antibacterial peptides—nisin.	In vitro	Hydrogel with cytocompatibility and good antimicrobial activity for wound dressing applications.	[94]
Xanthan gum	Hydrogels based on Aloe vera, xanthan gum, natural salicylic acid and allantoin.	In vitro	Natural, biocompatible hydrogels with antibacterial activity developed for dermatological applications.	[106]

**Table 3:** The main characteristics of polysaccharide-based hydrogels.

System	Characteristics of Biological Activity	Applications	Reference
Chitosan-fucoidan-(30%) pullulan-moxifloxacina	Delivery of moxifloxacin with constant release for 24 h.	Microneedle patches for rapid hemostasis and analgesia with bactericidal activity in wound healing.	[140]
Sodium alginate-gelatin-(6, 8, and 10 μmol/L) clindamycin	Transdermal delivery and fast release of clindamycin.	Microneedle patch for the treatment of acne with the prevention of the development of *C. acnes*.	[141]
(1.25%) Kappa-carrageenan-β-cyclodextrin- methotrexate	Methotrexate drug delivery.	Topical application	[142]
(10%) poly(vinyl alcohol) (PVA)-sulfated galactofucan (DF)(volume ratio of PVA:DF = 30:1, 20:1, 10:1, and 5:1)	Stimulates the recovery of the skin wound by obtaining a full-thickness layer.	Dual-functional hydrogel for repairing and regenerating diabetic skin ulcers.	[143]
(3%) Chitosan (CS)-fucoidan (FD)-(0.1%) triamcinolone acetonide (weight ratio of CS: FC = 7:3)	Oral mucosa patches with triamcinolone acetonide drug.	Hydrogel active in reducing inflammation and stimulating the formation of collagen fibers for diseases of the oral mucosa.	[144]
(0.1 g, 0.2 g and 0.4 g) of fraction isolated from the Periplaneta americana plant residue-(2.5%) carboxymethyl cellulose-(2%) carbomer 940	Topical dressings for the prevention and care of diabetic ulcers.	Hydrogel with wound healing potential in the diabetic rat model.	[145]
(2%) Glycyrrhizic acid-(2.5%) hyaluronic acid derivatives-(100 μg/mL) deferoxamine	Hydrogel with anti-inflammatory and angiogenic effects in the healing of burn wounds in rats, which promotes cell proliferation and migration.	Potential natural bioactive dressing based on herbal for burn wound management.	[146]
(2%) Chitosan-(0.1%) extract of flavonoids isolated from the leaves of Passiflora edulis Sims	In vitro and in vivo studies on antioxidant characteristics and healing of skin lesions in diabetic rats.	Dressings in the treatment of wounds.	[147]
(1-3%) Quaternized chitosan-(5%) tannic acid	In the model of skin defects in diabetic rats, the hydrogel promotes coagulation, suppresses inflammation and increases collagen deposition.	Injectable hydrogel dressing in clinical management of complex diabetic wounds.	[148]
(3%) Sodium alginate-(10%) polyvinyl alcohol-(3%) polycaprolactone microspheres	Effective scaffold to promote tissue regeneration and wound healing in a burn rat model demonstrated by in vivo studies.	Hybrid micro-sphere/hydrogel system for accelerated wound healing.	[149]
(2%) Quaternary ammonium chitosan-(2%) poly(tannic acid)-(2%) oxidized β-glucan	Natural hydrogel with a long-lasting bioeffect and improved mechanical properties through the introduction of poly(tannic acid) nanorods.	Hydrogel multifunctional dressings to accelerate the healing of complex diabetic wounds.	[150]
(1% and 2%) Chitosan-(0.5%, 1% and 1.5%) Curcumin	Incorporation of bioactive curcumin enhanced antibacterial ability of hydrogels for wound healing.	Wound healing applications.	[151]
(0.4, 0.8, and 1.2%) Pterocarpus marsupium heartwood extract-chitosan nanoparticles loaded hydrogel	Hydrogel with antimicrobial properties, efficient streptozotocin drug release and rapid wound healing action in diabetic rat models with re-epithelialization and granule tissue growth and collagen deposition.	Treatment for healing diabetic wounds.	[152]
(4%) Xyloglucan-(2%) poly(vinyl alcohol)	Hemocompatible film, partially adhesive, with antimicrobial action for *E. coli* in wound healing.	Wound healing applications.	[153]
(0.5, 1 and 1.5%) Pectic polysaccharide-derived from okra (*Abelmoschus esculentus* L. Moench) -based hydrogel	It improves wound healing by accelerating the regeneration of the dermis, inducing the formation of blood vessels and granulation tissues.	Effective dressings for the management of chronic diabetic wound healing.	[154]
